# Practice supporting system with related problem set generator based on targeted educational effects

**DOI:** 10.1007/s41039-015-0004-2

**Published:** 2015-06-23

**Authors:** Yasuhiro Noguchi, Satoru Kogure, Tatsuhiro Konishi, Yukihiro Itoh

**Affiliations:** 1 Faculty of Informatics, Shizuoka University, 3-5-1, Johoku, Naka-ku, Hamamatsu 432-8011 Japan; 2 Graduate School of Informatics, Shizuoka University, 3-5-1, Johoku, Naka-ku, Hamamatsu 432-8011 Japan; 3 Shizuoka University, 836, Ooya, Suruga-ku, Shizuoka 422-8529 Japan

**Keywords:** Problem generation, Intelligent educational systems, Related problem sets

## Abstract

Exercises with well-designed similar problem sets are effective in classrooms. In this case, teachers design similar problem sets related to the educational effects they have targeted. However, to design these “related problem sets (RPSs)” is not so easy for teachers, especially for students who are studying the problems. To support them, an intelligent tutoring system is expected to generate RPSs for teachers’ and learners’ targeting educational effects and support exercises for learners using these RPSs. It is useful for teachers who provide RPSs to learners with their educational effects and/or learners who want to study by themselves to get rid of their own weakness. This paper suggested the RPS generation and exercises supporting functions by an intelligent tutoring system for high school chemistry named Intelligent Practice Supporting System (IPSS). Some experiments confirmed that the performance of RPS generation and the exercises with IPSS had better educational effects than the ones without RPSs.

## Background

In classrooms, exercises constituted of multiple problems are often more effective than that of a single problem. For instance, exercises for knowledge stabilization can be constituted by many similar problems in which a common knowledge is used in the problem solving processes. Exercises for letting learners focus on the difference and/or commonness of two concepts can be realized by contrastive problems. We call such a set of problems, which has a certain educational effect “related problem set (RPS).” The educational effect of RPS depends on the combination of patterns in problems. In order to design RPS, teachers have to carefully choose appropriate problems, which have a certain relation to each other problem. For example, when a teacher wants to make a contrastive exercise for learners, he/she must choose contrast knowledge on themes of current learning and must make problems that such knowledge is essential to solve them. Even for a teacher, it is not easy to accomplish these tasks correctly, and it is especially difficult for learners who try to accomplish similar tasks. It is useful for teachers and/or learners that an intelligent tutoring system generates RPSs based on teachers’ and/or learners’ targeted educational effects and supports exercises with generated RPSs. In related works, some problem generation methods are suggested (Amuruth 2005; Martin and Mitrovic 2002). Nguyen-Thinh and Kojiri (2010) categorize the task of problem generation as the following two types. Our suggested Intelligent Practice Supporting System (IPSS) supports both of them:

- Approaches to derive solution structure as problem generation
- Approaches to derive surface structure as problem generation

Kojima and Miwa (2005) proposed a problem generation system supporting various problems by altering surface and structural features for mathematical word problems. Hirashima (Yamamoto et al. 2010; Hirashima et al. 2009) proposed a method to generate simplified problems for learners who fail to solve a difficult problem. Polya (1975) suggested that simplified problems are effective to help learners improve. The simplification is a part of RPSs supported by our IPSS. However, this research did not support to generate related problems based on teachers’ and/or learners’ target educational effects. It is inefficient to prepare whole patterns of related problems as learners can always try new problems; it is not effective enough to exercise related problems simply chosen from the same problem category. To give learners an environment where they can always try new related problem, RPSs should be generated based on the educational effects chosen by learners.

In previous research, we have already developed an intelligent tutoring system for high school chemistry named IPSS (Konishi et al. 2010). It had a solver unit, which can solve problems of high school chemistry (Okada et al. 2009), an adaptive explanation unit (Ishima et al. 2006), user interface for inputting answers, and other additional features. IPSS supports the following three types of problems:

A) Simulate a chemical phenomenon; a part of the result of the simulation is the answer.
B) Calculate a property value of a material using numerical relation knowledge.
C) Problems composed of A) and B).

In this paper, we extended this system to be able to generate RPSs based on teachers’ and/or learners’ target educational effects and support exercises with generated RPSs. First, we analyze the RPSs based on targeted educational effects by using a case study approach. Next, we explain the RPS generator of the extended IPSS and the functions of the extended IPSS for letting learners study with RPSs effectively. Then, we plan the experiments and report the results. Finally, we conclude this paper.

## Methods

### Related problem sets based on targeted educational effects

#### Case study on educational effects of RPS and relations among problems in a RPS

We use a case study approach to classify RPSs based on their expected educational effects. By analyzing collected problems from high school chemistry textbooks, we have found 16 problem groups, which can be regarded as RPS. In this paper, we chose eight problem groups in Table 1, because these problem groups could be based on knowledge of materials, phenomena, and calculation processes in the chemical world supported by our previous system. The remaining problem groups like memorization problems and history problems are omitted in this paper.

**Table 1:** Expected educational effects and methods of transforming

RPS type	Expected educational effects		Transforming method
	Effects	Method	
1	Making knowledge stable (target: general phenomenon knowledge)	Let learners use concrete phenomenon knowledge belonging to general knowledge repeatedly.	(iii)
2	Making knowledge stable (knowledge of numerical relationships)	Let learners use the knowledge of numerical relationships repeatedly.	(ii-1)
			(v)
3	Learning how to use knowledge of numerical relationships	Let learners apply the numerical relationship knowledge to various situations.	(ii-1)
			(ii-2)
			(v)
4	Making knowledge stable (knowledge of material concept)	Let learners use the knowledge of material concept repeatedly.	(ii-2)
5	Learning to apply conditions	Let learners become aware of the boundaries of applying conditions by using situations that can apply knowledge (positive example) and/or cannot apply it (negative example)	(iii)
			(iv)
6	Learning the hierarchy of material classes	Let learners become aware of material classes by finding the difference among problem solving processes in which a material belonging to the target class appears (positive example) and/or not belonging to the class (negative example).	(iii)
			(iv)
7	Increasing learners’ motivation to solve the problem	Making the problem easier with simplification.	(i) Deletion
8	Increasing learners’ ability for complications	Transitioning to advanced problems by making the problems more complicated.	(i) Addition

We analyzed the method of transformation from one problem to other problems in a RPS. In our previous system, there are two models to solve given problems: Chemical World Model (CWM) and Problem Solving Process Model (PSPM). CWM represents phenomenon in the chemical world of the problem by three states: before the chemical reaction, in the process of the chemical reaction, and after the chemical reaction. PSPM is a tree structure which represents calculation process in the problem. PSPM has three types of nodes: goal node, term node, and formula node. A goal node and term node are written by a chemical material name, their attributes, and their values. A goal node represents the calculation results of a problem or sub-problem. A term node is a member of the calculation. A formula node has a formula for the calculation and connects a goal node and a term node.

Our basic approach for generating similar problems in a RPS is to transform the representations of these models for a problem in the RPS. As a result, we found the following five typical transformation methods:

(i) Change of the number of steps of calculation by changing the goal or initial conditions (addition or deletion of calculation process).
  For example, when the original problem is “calculate the mass of 0.75H_2_O” by using a formula “mass = amount of substance × molar mass,” this problem is transformed to “calculate the mass of H_2_ in 0.75H_2_O” for adding a calculation step.
(ii) Change in the process of calculation by changing the goal or initial conditions.
  1.  Calculating the same (sub) goal by other knowledge as “A” using “A = B * C” → calculate “A” using “A = D/E.”
    For example, when the original problem is “calculate the mass concentration in the case that salt solution is 100 g and salt as solute is 1 g,” this problem is transformed to “calculate the mass concentration in the case that salt as solute is 1 g and water as solvent is 99 g.”
  2. Calculating another (sub) goal by the same knowledge as “A” using “A = X * C” → calculate “B” using “B = A/C.”
    For example, when the original problem is “calculate the mass of 0.75H_2_O” by using a formula “mass = amount of substance × molar mass,” this problem is transformed to “calculate the amount of substance of H_2_O whose mass is 13.5 g” by using a formula “amount of substance = mass ÷ molar mass.”
(iii) Change materials without changing general phenomenon knowledge; this is used for simulating phenomenon on the problem.
  For example, if the original problem is “2H_2_ + O_2_ → ?” by using knowledge of the combustion reaction, this problem is transformed to “CH_4_ + 3O_2_ → ?” by using the same knowledge.
(iv) Change materials by changing general phenomenon knowledge; this is used for simulating phenomenon on the problem.
  For example, when the original problem is “2Cu + O_2_ → ?” by using knowledge of the combustion reaction, this problem is transformed to “CuO + H_2_ → ?” by using knowledge of the reduction reaction.
(v) Simple change only on numerical value included in initial conditions.
  For example, when the original problem is “calculate the mass of 0.75H_2_O” by using a formula “mass = amount of substance × molar mass,” this problem is transformed to “calculate the mass of H_2_O” by using the same formula.

Table 1 shows the relationships among the expected educational effects, the RPS, and the method of transformation from one problem to other problems in a RPS. In the next section, we would like to focus on the details of types “5” and “6” in Table 1 as they are more complex than the others.

#### Learning to apply conditions and the hierarchy of material classes by cognitive conflict

In the high school chemistry area, applying conditions for knowledge is defined by the boundary for the condition whether the knowledge is available or not. Through exercises that could be solved by using a specific knowledge and/or other knowledge, learners can find the boundary by comparing the conditions of each exercise.

Material classes are an important element of the boundary definition. Applying conditions for knowledge is not written for every specific material. It is written by using bounding material classes. The hierarchy of material classes represents inclusion relationships among material classes, such as Fe being a member of the metallic class. The metallic class is also a member of chemical class. Learning the hierarchy of material classes is essential for understanding the applicability of each material in applying conditions.

To sum up the exercises for learning both applying conditions and the hierarchy of material classes, the exercises should let learners focus on the differences and commonness of conditions between the problems where knowledge can be used (positive examples) and cannot be used (negative examples). We expect this learning method to be more effective than the method that a teacher gives learners by applying conditions and/or inclusion of relationships among materials explicitly, as would allow learners to discover the boundary within fundamental thinking and the differences and/or commonness among examples.

**Fig. 1:**
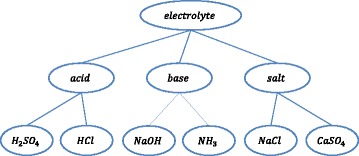
Hierarchy of material classes

When learners learn by using positive and negative examples, they can learn more effectively from their cognitive conflict. Cognitive conflict means a conflict caused from the differences between the learner’s existing beliefs and correct knowledge. Learners often feel cognitive conflicts when they cannot find correct answers. Cognitive conflict causes learners to acquire new knowledge which is useful for resolving the conflicts (Inagaki and Hatano 1971). We can design RPSs for inducing learners’ cognitive conflicts that have arisen from their misunderstanding of applying conditions and/or material to classes. As an example of such RPS, there is a knowledge (named K1) whose applying condition is “If *x* ∈ acid”; a hierarchy of material classes related with K1 is shown in Fig. 1. Suppose a learner has misunderstood the condition of K1 as “If *x* ∈ electrolyte.” He has solved an original problem on HCl (it belongs to both acid and electrolyte groups) using K1, successfully. In such a case, we can cause cognitive conflict by using a problem that is generated by replacing HCl with NaCl (it belongs to the electrolytes, but *not* to acids) in the original problem. The learner will apply K1 to the generated problem; then he/she will feel some cognitive conflict from the problem. We show methods of changing an original problem to such RPS by each pattern of misunderstanding in Table 2.

**Table 2:** Patterns of misunderstandings and methods of changing materials (assume H_2_SO_4_ appears in the original problem)

Target of learning	Pattern of misunderstanding	Example	Changing materials
Applying conditions (e.g., *x* ∈ acid)	Applying conditions understood by learners who are narrower than the correct one.	If *x* ∈ H_2_SO_4_	H_2_SO_4_ → HCl
	Applying conditions understood by learners who are broader than the correct one.	If *x* ∈ electrolyte	H_2_SO_4_ → NaCl
Hierarchy of material classes	In learners’ understanding, a material belongs to a class, although, it does not in fact, belong.	NaCl ∈ acid	H_2_SO_4_ → NaCl
	In learners’ understanding, a material does not belong to a class, although it does, in fact, belong.	HCl ∉ acid	H_2_SO_4_ → HCl

### Related problem set generator

#### Basic procedure of related problem generation

Our RPS generator is premised on three inputs: “an original problem,” “expected educational effects (seen in Table 1),” and knowledge that learners should learn with the RPS (named “target knowledge”). The RPS generator transforms PSPM and CWM of the original problem to PSPM and CWM of its RPS based on the expected educational effects (see the column “Transforming method” in Table 1). The details of the transformation for each educational effect are discussed in the next section. After the transformation, to ensure the consistency of the whole transformed problem, the RPS generator propagates the modification on the CWM to the PSPM and vice versa. After these processes, the RPS generator extracts the goal and initial conditions of the generated problem; then, the RPS generator translates its CWM and PSPM into its problem text by using templates.

#### Method of transforming PSPM and CWM according to expected educational effects

##### (1) RPS type 1: making general phenomenon knowledge stable

To change materials without changing the general phenomenon knowledge in the original problem, the RPS generator retrieves concrete phenomenon knowledge of the target knowledge at random. It swaps the concrete phenomenon knowledge on the CWM of the original problem to the retrieved concrete phenomenon knowledge.

##### (2) RPS type 2: making knowledge of numeral relation stable

To change numerical relationships without changing the target knowledge in the original problem, the RPS generator searches the target knowledge from the PSPM of the original problem. The RPS generator changes both the higher nodes in the PSPM than the target knowledge and the lower ones. It retrieves the knowledge of numerical relations suitable for swapping with a formula in the changed nodes.

##### (3) RPS type 3: learning how to use the knowledge of numerical relationships

The RPS generator searches the target knowledge from PSPM and transforms the formula of the target knowledge by transposing a term. It appends some nodes of the PSPM to lower and higher places than the node of the transformed target knowledge. It generates added nodes by retrieving suitable knowledge of numerical relationships, by a similar way as (2).

##### (4) RPS type 4: making knowledge of material concept stable

To generate RPS by calculating the other (sub) goal with the same knowledge as that of the original problem, the RPS generator searches a node representing a property value of the target material in the PSPM. It then focuses on the formula attached to the node and changes the PSPM without the removal of the focused formula at random (using the same methods as (2) and (3)).

##### (5) RPS type 5: learning to apply conditions

The RPS generator should generate problems for “learning by positive and negative examples” as mentioned in the “Learning to apply conditions and the hierarchy of material classes by cognitive conflict” section. In order to generate positive examples, the RPS generator changes the CWM and PSPM of an original problem by keeping the application of conditions to the target knowledge. In case the applying condition is written by a phenomenon, the transformation is performed the same way as in (1). In case the applying condition is written for a material class, the RPS generator replaces the concrete material with another concrete material, which can then satisfy the applying conditions of the target knowledge. On the other hand, to generate negative examples, the RPS generator changes phenomenon or the material of the applying condition to other ones that cannot satisfy the applying condition. In this transformation, the RPS generator should replace the phenomenon or the material with a similar one as often as possible, as it will show learners the boundaries more clearly.

##### (6) RPS type 6: learning the hierarchy of material classes

This method is very similar to (5). The RPS generator also generates problems as positive and negative examples. To generate a positive example, it swaps a material in the CWM and PSPM with another material that belongs to the same material class. To generate a negative example, it swaps a material with another material that does not belong to the material class, with the consideration for choosing a similar material as often as possible.

##### (7) RPS type 7: supporting problem solving with simplification

The RPS generator searches the target knowledge from the PSPM or the original problem. It deletes some nodes from the PSPM while keeping the target knowledge.

##### (8) RPS type 8: raising learners’ ability by the use of complications

The RPS generator appends some nodes into the PSPM of an original problem. These nodes are at a lower place than leaf nodes and/or a higher place than the goal node. The method to generate additional nodes is the same as (3).

#### Procedure following the changing of CWM and PSPM

##### Modification after changing CWM

After changing the CWM of an original problem, the RPS generator needs to modify the PSPM, if the original problem has its PSPM (in the case of the transforming method (iii)). The RPS generator modifies the materials in the PSPM, if some materials in the CWM have been changed. In addition, the RPS generator checks whether applying conditions to all knowledge of numerical relationships are satisfied under the modified CWM. If by applying conditions to knowledge have not been satisfied, that knowledge is then replaced by other available knowledge. The available knowledge is then searched by the same method used in RPS type 2, the transforming PSPM and CWM method.

##### Modification after changing PSPM

After changing the PSPM of an original problem, the RPS generator needs to modify the CWM. If the transformation of the PSPM added knowledge of numerical relation, the RPS generator modifies the phenomenon in the CWM to satisfy the applying condition of the added knowledge.

##### Generating a problem from modified CWM and PSPM

As mentioned in the “Background” section, IPSS can handle three types of problems. The RPS generator knows which part of CWM and PSPM should correspond to the goal and initial conditions, for each type of problem. The leaf nodes of PSPM become initial conditions, and the root node becomes the goal of types A) and B) of problems. Reactants appearing in CWM become initial conditions of types A) and C) of problems. And the RPS generator sets the goal of type A) of problems to the same part as the original problem (e.g., chemical formula of product). The RPS generator describes the initial conditions and the goal using the grammar we developed for problem representations. Then, the RPS generator translates the problem representations into the text by using templates.

#### Overview of the RPS generation in our system

**Fig. 2:**
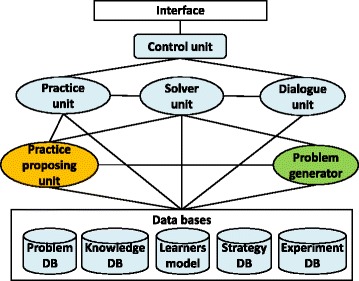
Overview of the system

IPSS is developed using Java language and works on Microsoft Windows. The extensions in the paper are the “problem generator (RPS generator)” and the “practice proposing unit (PPU)” in Fig. 2. After a learner finishes solving a problem, the PPU proposes additional practice with related problems generated by our RPS generator. Targeted educational effects used for the problem generation is set by teachers beforehand or set by IPSS in order to treat learner’s weak points. For instance, if a learner could not handle a phenomenon knowledge at the beginning, the exercises in which the learners would find how to use the phenomenon knowledge would be proposed by IPSS, and the exercises include RPS type 1 (making a knowledge stable (target: general phenomenon knowledge)) generated by the RPS generator.

#### Example of related problem generation

**Fig. 3:**
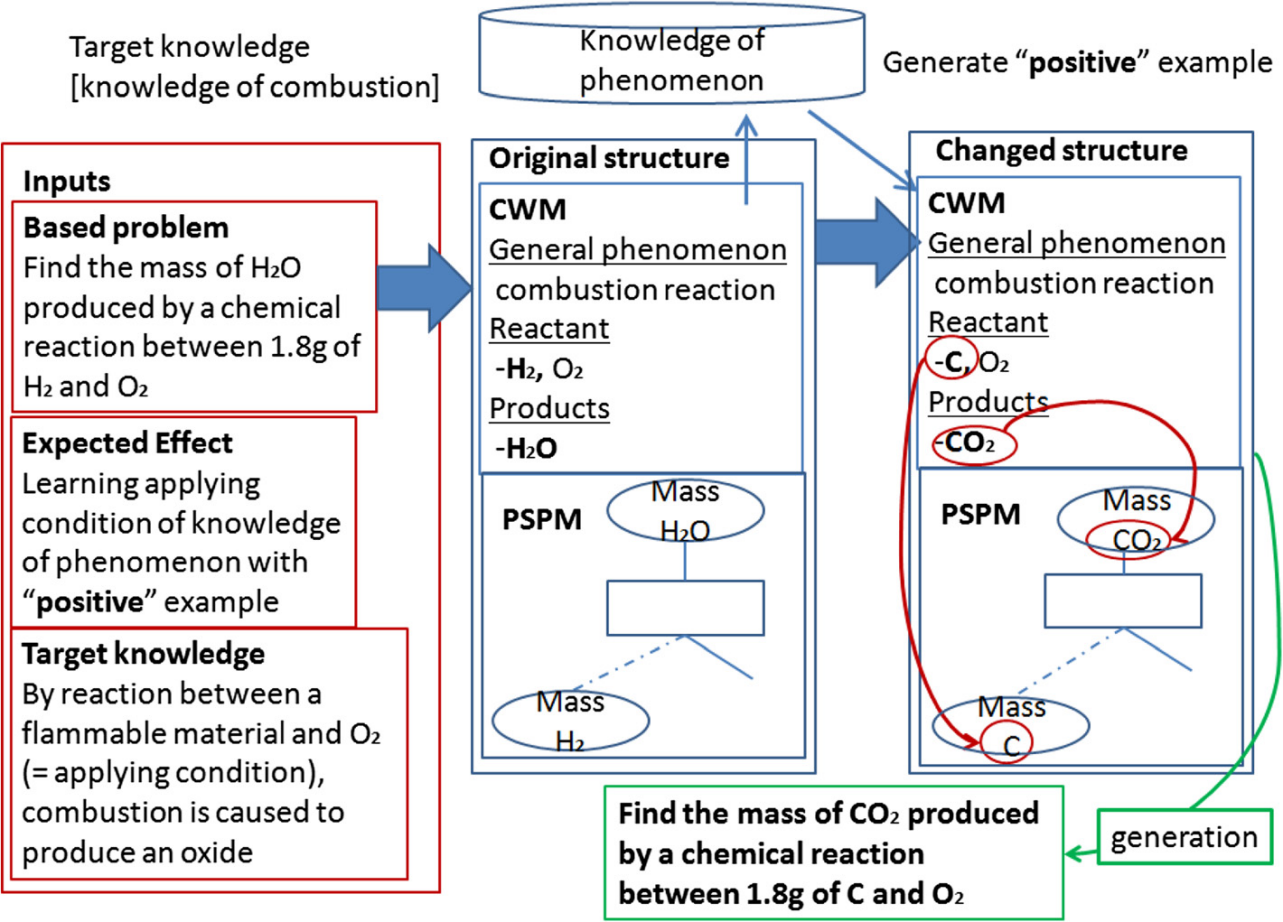
Example of related problem generation

Figure 3 shows an example of the generating process of a related problem. In this example, the original problem is “Find the mass of H_2_O produced by a chemical reaction between 1.8 g of H_2_ and O_2_,” the targeting educational effect is “Learning applying condition of knowledge of phenomenon,” and the target knowledge is general phenomenon knowledge that “By reaction between a flammable material and O_2_ (= applying condition), combustion is caused to produce an oxide.” The system applies the procedure for RPS type 5 (5) in the previous section to the CWM. The applying condition is a combination of “a flammable material” and “O_2_.” They correspond to “H_2_” and “O_2_” in the original problem. They can be changed to another combination which also satisfies the applying condition. In order to generate a positive example, we retrieve a concrete phenomenon knowledge belonging to the target general phenomenon knowledge. Our current system chooses a knowledge from retrieved candidates at random. However, considering effective learning on boundary of applying conditions, it may be better to choose the candidate including a material which has the maximum distance in the hierarchy of material classes from the material in the original problem. In this example, we assume the system chooses the chemical reaction between C and O_2_. The RPS generator changes the CWM using this knowledge of phenomenon.

By the procedure after changing CWM and PSPM in the previous section, the system replaces “mass of H_2_O” as the goal in the PSPM with “mass of CO_2_,” because the transforming process changed the original product “H_2_O” to “CO_2_” in the CWM. After that, the system generates a problem from modified CWM and PSPM. The RPS generator extracts initial conditions and the goal from modified CWM and PSPM, to generate a problem “Find the mass of CO_2_ produced by a chemical reaction between 1.8 g of C and O_2_.” By letting a learner solve the original problem and the generated one successively, he/she can perform practice effective on understanding the applying condition of combustion.

#### Supporting function for effective study with related problem sets

We classified essential functions for supporting learners’ exercises using RPS to the following two types:

- Function (a): functions for inducing learners to exercises using RPS
- Function (b): functions for letting learners focus on the target knowledge of the exercise using RPS

The function (a) supports learners to naturally shift to RPS-based exercises. In general, learners start from a single problem exercise. After that, the learners should try new exercises using RPS based on educational effects suited for the learners. We think two types of guides should be available. One is that learners consciously choose a RPS by its targeted knowledge and its educational effects. The other is that learners ask an intelligent tutoring system to choose an adequate RPS for the learners based on their solving problems. The function (a-1) supports the former, the function (a-2) supports the latter, and the function (a-3) supports learners who could not solve their single problem exercise.

The function (b) supports learners to study on RPS-based exercises effectively. Exercises using a RPS work more effectively when the learners focus on the target knowledge in the RPS, than when the learners just solve the problem. We proposed two functions based on these ideas. One is that suggesting important points should be focused during the exercise. The other is that learners should be supported to observe problems contrastively.

#### Function (a): functions for inducing learners to exercises using RPS

##### (a-1) Function of preparing exercises using RPS designed by teacher

In IPSS, teachers can generate RPSs by using the RPS generator and register generated RPSs into their RPS database. When a teacher uses the RPS generator, he/she can choose RPS types by their targeted educational effects from Table 1. The learners are given a list of RPSs prepared by the teacher, which shows the target knowledge, targeted educational effects, and the problem description. By using the list, the learners can consciously choose RPS with consideration for the targeted knowledge and adequate educational effects for them.

##### (a-2) Function of preparing problems with knowledge that learners cannot use correctly

IPSS diagnoses the learners’ answers in exercises. The answers are analyzed by using the CWM and PSPM of the problems, and learners’ misunderstanding and/or unstable knowledge issues are discovered. By working this function, learners can ask IPSS to generate exercises using RPS that has educational effects for the learners’ weak points. IPSS calls the RPS generator and decides essential inputs in order to prepare exercises using the RPS for the learners. In particular, a problem the learner cannot solve is set as “an original problem”; discovered learners’ misunderstanding and/or unstable knowledge is set as the “target knowledge”; and the “expected educational effects” is chosen from RPS types 1, 2, and 4 (“making knowledge stable”) in Table 1 and depends on the types of discovered learners’ misunderstandings and/or unstable knowledge issues.

##### (a-3) Function of preparing easier problems than original problems

Some learners are at an impasse with solving original problems. An intelligent tutoring system that supports the RPS generation can help these learners by preparing simple versions of the problem learners try to solve. IPSS has prepared an interface to accept not only learners’ answer but also the intermediate state of their problem solving. It enables IPSS to diagnose their impasse and find knowledge the learner cannot handle. IPSS decides the problem the learners cannot solve to “an original problem” and assigns knowledge the learners cannot handle to the “target knowledge,” as well as chooses “supporting problem solving with simplification” (RPS type 7 in Table 1) as the “expected educational effects.”

The generated problem is very simple in that it can be solved by using the target knowledge. It is expected that the learners remember the correct method to use the target knowledge through solving the simple problem. After the learner can solve the simple problem, IPSS shows a message to suggest the original problem can be solved in the same way, in order to let learners become aware that the simple problem is part of the original problem. When this function is executed, IPSS stores the current status of the problem solving. It is enabled to restore the status of the process of solving the original problem and to let learners retry the original problem.

#### Function (b): functions for letting learners focus on the target knowledge of the exercises using RPS

##### (b-1) Function for suggesting target knowledge

For learners who have an exercise using RPS, the suggested important points should be focused throughout the exercise. To focus on the target knowledge of the RPS, the suggested important points are generated according to the targeted educational effects by the teachers and/or the learners. Table 3 shows message templates for each RPS type. Moreover, the messages that let learners be reminded of the target knowledge in the problem are given to the learners after the learner succeeds in solving the problem (in Table 4). The blanks ([ ]) of the templates are filled with words retrieved from the CWM or PSPM of the RPS (These messages are translated for this paper. The original messages are written in Japanese).

**Table 3:** Messages generated during an exercise

RPS type	Message
1	You should pay attention to the combination of reactants in the phenomenon of [Name of phenomenon].
2	You should pay attention to general relationships among [Property1]…[Property n].
3	You should pay attention to the numerical relations; [Formula].
4	Learn [Property] of [Class of Material].
5	You should pay attention to the conditions for applying knowledge to a problem.
6	You should pay attention to both commonness and differences among the natures of materials.

**Table 4:** Messages generated after an exercise

RPS type	Message
1	In this problem, [Names of reactants] react and [Names of phenomenon] happen.
2	In this problem, [Property] is calculated by using [Formula].
3	In this problem, [Property] is calculated by using [Formula].
4	In this problem, you have used that [Property] of [Class of Material] is [Value].
5	In this problem, you can use knowledge as these conditions are satisfied; [condition 1]…[condition n].
6	In this problem, it is important that [Class 1 of Material] belongs [Class 2 of Material].

##### (b-2) Function of explaining differences among problems

For supporting learners to observe problems contrastively, IPSS shows the difference between the original problem and the exercised problem in the RPS. IPSS contrastively shows the following information for each problem: each problem description, each answer, explanation of each problem solving process, and important points to be focused on in each problem (this is also generated by templates in Table 3).

## Results and discussion

### Experiments

#### Experiments for related problem set generation

The effective exercises using RPSs is based on the performance of RPS generation. The purpose of the experiment is to confirm the performance of RPS generation before the experiments for confirming the educational effects of the exercises by using RPSs. We think the important indexes of the performance of RPS generation for teachers and/or learners as being “problems in the generated RPS appropriate for the teachers’ or learners’ targeting educational effects or not” and “text explaining the generated problems are possible for use by learners.”

In these experiments, the subjects evaluate the problems generated by the RPS generator. We requested the subjects do two evaluations. One being whether “the generated problems are appropriate for the targeting educational effects or not.” The other is the “text explaining the generated problems are possible to use for learners.” The evaluation was defined in 5 levels, with 5 being good, 3 being neutral, and 1 being bad.

The subjects are ten students—both university and graduate school students, whose main subject area is informatics. All of them learnt chemistry in high school. We gave the subjects related problems with their original problems, the answers of the original problems, essential knowledge needed to solve the original problems, and targeting educational effects (explanations of RPS type) of exercising the related problems. These related problems were generated by our system based on the targeting educational effects.

This experiment prepared ten generated problems. RPS types (in Table 1) and problem types (mentioned in the “Background” section) as each generated problem shown in Table 5. Four RPS type 5 problems were prepared in the ten generated problems as “learning applying conditions” type; RPS type 5 is one of them and is needed to evaluate both positive and negative examples. As for the negative example, these problems included a negative example in which the target knowledge could not work. This composition included all problem types, and we prepared many problems of type C) because our problem set generation was designed that its generation process for type C) is a combination of the generation process for types A) and B).

**Table 5:** Composition of the generated problems for evaluation

	1	2	3	4	5	6	7	8	9	10
RPS type	2	5	5	5	5	1	6	2	2	3
Problem type	B	A	A	C	C	C	C	C	C	C

#### Experiments for effective study with related problem sets

The purpose of the experiment was to preliminarily confirm whether the related problem generated by our system could be effectively used in a study with RPS or not. The subjects are eight university and graduate school students, whose main subject is informatics. All of them learnt chemistry in high school. It might be better if the subjects were high school students. However, IPSS is designed for reviewing after teachers, so the subjects should have a certain level of knowledge in chemistry. In this sense, the subjects are tolerable.

We performed pre-tests in order to group the subjects into an experimental group (group E) and a control group (group C); each of which had comparable scores. First, we gave them 20 min of instructions in using the IPSS. Secondly, both of the groups practiced solving chemical problems for 30 min. Subjects in group C used old IPSS that could not handle RPS. They were given a compulsory problem and a problem list. After they solved the compulsory problem, they could solve any problem from the list as they wished. Subjects in group E used extended IPSS, which has the RPS generator and extended functions as mentioned above. They were then given a compulsory problem, a generated problem, and the problem list. The compulsory problem and the generated problem composed of an RPS for targeting educational effects is “learning applying conditions.” We chose the related problem as the problem was generated by the most complex generation process in all of RPS types in our system. After they could solve the RPS problems, they could solve any problems in the list as they wished.

The following problems for their exercises were given to the subjects:

- [Compulsory problem (for both of the groups)]
- 2.3 g of K and HCl react, and then H_2_ gas is produced. Find the math of the H_2_.
- [Generated problem (for only group E)]
- 6.4 g of Cu and H_2_SO_4_ react, and then H_2_O is produced. Find the math of the H_2_O.

Notice that the chemical reactions occurring in these two problems do not belong to the same class. Usual metals such as K and acid react, and then H_2_ gas is produced. But Cu has a lower ionization tendency than H. Such metals and acid do not react in such a way. Only very strong acids with such metals react, and then H_2_O is produced. Such differences are very important in applying conditions of knowledge on these chemical reactions. Finally, we performed post-tests, which had the same questions as the pre-test.

### Results

#### Results for related problem set generation

Table 6 shows the appropriate rate of the generated problems for targeted educational effects. The highest average rate is 5.0 for problem 1. The lowest average rate is 4.0 for problem 5. The average rate for ten problems is 4.6. The total average score confirmed that the RPS generator could generate RPS based on the targeted learning effects. And the lowest average score shows the RPS generator could be used for these RPS types and problem types.

**Table 6:** Appropriate rate of the generated problems for the targeting educational effects

Problem	1	2	3	4	5	6	7	8	9	10	Total
Average	5.0	4.5	4.2	4.8	4.0	4.6	4.8	4.6	4.2	4.9	4.6

The low average rate problems (problems 3, 5, and 9) are designed as negative examples in which the target knowledge could not work. In the questionnaires, some subjects explained that as being the reason for a low score for the problem. One subject pointed out that IPSS should have announced to learners that a problem in which the target knowledge could not work possibly existed in the generated problem.

Table 7 shows the possibility rate of the explaining text to use for learners. The highest rate is 5.0 for problems 1 and 7. The lowest rate is 4.2 for problem 3. The total average rate for ten problems is 4.8. The total average rate and the lowest rate confirmed the text explaining the generated problems by the RPS generator that could be used for learners in classes. In questionnaires, subjects requested that the style of surface expressions should be formalized between the original problem and the generated problems.

**Table 7:** Possibility rate of the explaining text to use for learners

Problem	1	2	3	4	5	6	7	8	9	10	Total
Average	5.0	4.5	4.2	4.7	4.7	4.9	5.0	4.9	4.7	5.0	4.8

#### Results for effective study with related problem sets

Table 8 shows the scores of the pre-test and the post-test. The improvement of the experimental group seems to be better than the control group. It suggests a certain educational effectiveness of our extended IPSS. However, we should perform qualitative analysis as to why group E improved more than group C. We focused on questions of the reaction between acid and metal, because the RPS given to group E handles such reactions. Such types of questions in the pre-test and post-test are seen in Table 9. It also shows improvement from the practice of each group.

**Table 8:** Result of pre-test and post-test

	Control group			Experimental group		
Subject	Pre-test	Post-test	Improvement	Pre-test	Post-test	Improvement
1	6.6	6.8	0.2	7.2	7.6	0.4
2	4.9	5.1	0.2	6.4	9.6	3.2
3	4.5	4.3	−0.2	6.0	9.2	3.2
4	3.4	6.0	2.6	3.3	4.4	1.1
Average (S.D.)	4.9 (1.15)	5.6 (0.94)	0.7 (1.11)	5.7 (1.47)	7.7 (2.05)	2.0 (1.25)

**Table 9:** Improvement of scores of questions on chemical reaction between acid and metal

	Number of correct answers pre-test/post-test (gain)	
	Group C	Group E
Q-A) Describe the reaction formula when Ca and H_2_SO_4_ react.	2/2 (+0)	2/2 (+0)
Q-B) Describe the reaction formula when Cu and H_2_SO_4_ react.	0/0 (+0)	*0/3 (+3)*
Q-C) In general, how do acids and metals react?	0/0 (+0)	*0/2 (+2)*
Q-D) Do the following combinations of materials cause chemical reactions?		
(a) Na and H_2_SO_4_	3/4 (+1)	4/4 (+0)
(b) Cu and HCl	1/1 (+0)	1/2 (+1)
Q-E) Do the following combinations of materials cause the production of H_2_ gas?		
(a) Pb and HCl	2/3 (+1)	3/4 (+1)
(b) Ag and H_2_SO_4_	1/3 (+2)	0/2 (+2)

Improvement on questions Q-B) and Q-C) suggests the effects of the RPS given to group E. More than half of the subjects improved their scores, while no subject in group C did. We think that the RPS allows subjects to be aware that there are two kinds of chemical reactions between acids and metals, as mentioned above. Probably, the subjects in group C forgot the condition of the ionization tendency of metal. Therefore, they describe a wrong reaction formula for question Q-B), and they cannot describe the correct conditions of the reaction for question Q-C). In Q-C), only group E answered by using knowledge of the ionization tendency of materials. Some subjects in group E categorized metals based on the ionization tendency in their answers. The other subjects, these subjects did not care the ionization tendency of materials in the pre-test, did not use such category, but they calibrated their answers to exclude the reaction with only high ionization tendency metals. As a consequence, we find an exercise using RPS has certain educational effects, so the extended functions for handling RPSs improve the effectiveness of IPSS.

## Conclusions

We proposed an intelligent tutoring system that can design similar problem sets related to teachers’ and/or learners’ targeted educational effects. Our proposed intelligent tutoring system named IPSS has extended to generate such a related problem set (RPS) and supported learners in their exercises by using generated RPSs. In this paper, we proposed eight related problem sets categorized by their expected educational effects for high school chemistry and RPS generation methods by these eight categories. Our suggested functions, which support effective RPS-based exercises, can induce learners to practice exercises using RPS and let learners focus on the target knowledge of the exercise using RPS. Our experiments for the RPS generation confirmed that the performance of the RPS generation by extended IPSS, whether extended IPSS, can generate RPSs based on targeting educational effects and had been developed to a practical level. Furthermore, our experiments for effective study with RPS shows exercises with RPSs using extended IPSS had better educational effects than the ones without RPSs.

The current system has already supported approximately 50 % of the problems in the inorganic chemistry section in a high school chemistry textbook. Unsupported problems were categorized in memorization problems, problems using figures, history problems, and essay style problems. As a part of future works, IPSS should be evaluated in high school classes.
